# miR-200b inhibits proliferation and metastasis of breast cancer by targeting fucosyltransferase IV and α1,3-fucosylated glycans

**DOI:** 10.1038/oncsis.2017.58

**Published:** 2017-07-10

**Authors:** Q Zheng, X Cui, D Zhang, Y Yang, X Yan, M Liu, B Niang, F Aziz, S Liu, Q Yan, J Liu

**Affiliations:** 1 Department of Biochemistry and Molecular Biology, Dalian Medical University, Liaoning Provincial Core Lab of Glycobiology and Glycoengineering, Dalian, People’s Republic of China; 2 Department of Clinical Laboratory, The First Affiliated Hospital of Dalian Medical University, Dalian Medical University, Dalian, People’s Republic of China; 3 Department of Oncology, First Affiliated Hospital of Dalian Medical University, Dalian, People’s Republic of China

## Abstract

Aberrant protein fucosylation is associated with cancer malignancy. Fucosyltransferase IV (FUT4) is the key enzyme catalyzing the biosynthesis of α1,3-linkage fucosylated glycans carried by glycoproteins on the cell surface, such as the tumor-associated sugar antigen Lewis Y (LeY). An abnormal increase in the levels of FUT4 and LeY is observed in many cancers and correlated with cell proliferation and metastasis. Some microRNAs (miRNAs) are known to negatively regulate gene expression. FUT4 is an oncogenic glycogene, and thus it is important to identify the specific miRNA targeting FUT4. In current study, we first identified miR-200b as a specific miRNA that inhibited FUT4 expression. We found that miR-200b level was decreased, whereas that of FUT4 was increased in tissues and serum of breast cancer compared with that in the control by real-time PCR, western blotting and enzyme-linked immunosorbent assay. The alterations of miR-200b and FUT4 level were recovered after chemotherapy. The results also showed that miR-200b suppressed FUT4 expression and inhibited tumor growth and metastasis in MCF-7 and MDA-MB-231 breast cancer cells, as well as in the xenografted tumor tissues and metastatic lung tissues. miR-200b decreased the α1,3-fucosylation and LeY biosynthesis on epidermal growth factor receptor (EGFR), as well as inactivation of EGFR and downstream phosphoinositide-3 kinase/Akt signaling pathway. In conclusion, the study highlights that FUT4 could apply as a novel target for miR-200b that suppress the proliferation and metastasis of breast cancer cells by reducing α1,3-fucosylation and LeY biosynthesis of glycoproteins. miR-200b and FUT4 are potential diagnostic and therapeutic targets for breast cancer.

## Introduction

Breast cancer, one of the most frequent malignancies in women, is threatening women’s health. Its incidence and mortality are increasing,^1^ with approximately 130 000 new cases and 40 000 deaths per year in China.^2^ Although early-stage breast cancer shows relatively better outcomes after surgery and chemotherapy, approximately 90% of breast cancer deaths result from recurrent and distant metastasis of the primary tumor.^3^ Therefore, identifying early prognosis and therapeutic biomarkers may increase the survival rate of these patients.

Glycosylation is one of the most common posttranslational events in mammalian cells and influences the physical and chemical properties, as well as structures and functions of proteins.^4^ Cancer glycobiology studies have revealed that abnormal glycosylation is correlated with cancer proliferation, metastasis, pathological stages and prognosis.^5, 6, 7^ Protein fucosylation is considered as a characteristic alteration in tumorigenesis.^8^ Fucosylation modification of glycoproteins is catalyzed by specific fucosyltransferases (FUTs).^9^ Till now, 13 FUT family members have been identified. Fucosyltransferase IV (FUT4), one of the α1,3-fucosyltransferase, catalyzes the transfer of fucose from the donor of GDP-Fuc to form α1,3-linkage fucosylated glycan epitopes on the sugar chains of glycoproteins.^10^ For example, tumor-associated carbohydrate antigen Lewis Y (LeY) is a difucosylated oligosaccharide containing α1,2- and α1,3-linkage.^11^ Elevated FUT4 and its synthetic product LeY have been observed in many cancers, such as breast cancer, lung cancer and melanoma.^12, 13, 14^ In our previous study, we found that FUT4 functions as an oncogenic glycogene, as reported earlier.^13, 15^ Thus inhibition of FUT4 by miR-200b is a potential strategy for tumor malignancy suppression. Here we aimed to find the specific microRNA (miRNA) targeting FUT4 to inhibit cancer proliferation and metastasis.

miRNAs, classes of endogenous and small non-coding RNAs, consist of 21–23 nucleotides. The sequences of miRNAs are highly conserved and specific in the blood and some tissues.^16^ miRNAs downregulate the expression of target genes at the posttranscription level by binding to their 3′-untranslated region (3′-UTR), inducing translation inhibition or mRNA degradation. miRNAs are involved in some physiological and pathological processes, including embryo development, inflammation and tumorigenesis.^17, 18, 19^ The family of miR-200 includes five members: miR-200a, miR-200b, miR-200c, miR-429, and miR-141.^20^ miR-200b, which acts as an antioncogene, participates in the proliferation and metastasis inhibition of different kinds of cancers by downregulating target molecules. For instance, miR-200b inhibition promotes Rac1 activation and increases the metastatic potential of HBEC cells.^21^ miR-200b can repress angiogenesis by targeting angiogenic factors and receptors.^22^ It can inhibit the epithelial to mesenchymal transition (EMT) by inactivating transcription factors in breast cancer.^23^ miR-200b is associated with the estrogen receptor status of breast cancer cells.^24, 25^ Few studies have examined the regulation of fucosyltransferase expression by miRNAs. Previous studies reported that FUT8 level in spontaneous hepatocarcinoma was downregulated by miR-26a, miR-34a, miR-455-3p and miR-122.^26, 27^ Whether miR-200b suppresses the proliferation and metastasis of breast cancer cells via targeting FUT4, thus decreasing α1,3-fucosylated glycan biosynthesis of the glycoproteins, remains unclear.

Current methods for breast cancer diagnosis mainly depend on serological (cancer antigen 15-3, carcinoembryonic antigen, tissue polypeptide antigen, tissue polypeptide-specific antigen) and pathological observations.^28^ However, cancer antigen 15-3 and carcinoembryonic antigen show low positive rates (15%) for the early diagnosis of breast cancer and are not recommended by the American Society of Clinical Oncology and European Society for Medical Oncology. Therefore, it is crucial to identify specific and sensitive novel serum markers for clinical diagnosis, target therapy and treatment evaluation of breast cancer. Studies have shown that specific miRNAs can be used as potential markers for tumor diagnosis.^29^ Circulating miRNAs in the peripheral blood directly reflect coincident alterations of miRNAs in the corresponding tissues.^30^ Furthermore, circulating miRNAs are stable, convenient for detection and cause low trauma in patients.^31^

In the current study, we detected alterations of the levels of miR-200b and FUT4 in tissues, as well as in the serum of breast cancer patients, and found that the level of miR-200b is negatively correlated with FUT4 expression in breast cancer patients. We also showed that miR-200b promotes the proliferation and metastasis of MCF-7 and MDA-MB-231 cells by targeting FUT4 and α1,3-fucosylated glycans both *in vitro* and *in vivo*. Our results provide new information for serum diagnosis using miR-200b and FUT4 as key markers and therapeutic targets for breast cancer.

## Results

### miR-200b is negatively correlated with FUT4 in human breast cancer tissues and serum

**Figure 1:**
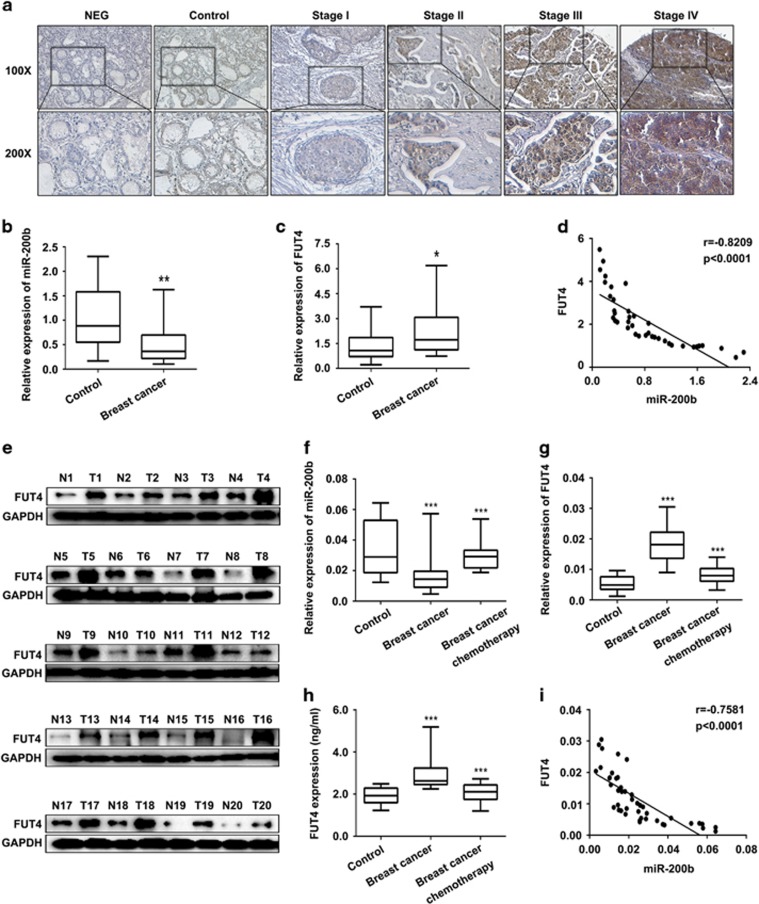
Low miR-200b and high FUT4 level in breast cancer patient specimens. (**a**) Detection of FUT4 expression in breast cancer tissue chip (100 cases, pathological grading from stage I to stage IV). Representative images were shown. NEG: PBS was used to replace primary antibody of FUT4. Control: normal human breast tissue. (magnification, × 100 and × 200). (**b**, **c**) Real-time PCR for miR-200b and FUT4 levels in tissues of breast cancer (T1-20) and matched adjacent non-cancerous normal tissues (N1-20). (**d**) The correlation between miR-200b and FUT4 levels in breast cancer and matched normal adjacent tissues. (**e**) Western blotting analysis for the expression of FUT4 in breast cancer tissues (T1-20) and matched adjacent non-cancerous normal tissues (N1-20). GAPDH served as an internal reference. (**f**) Expression of miR-200b in serum of healthy controls, breast cancer patients and patients after chemotherapy was detected by real-time PCR. (**g**, **h**) Real-time PCR and enzyme-linked immunosorbent assay for the level of FUT4 in serum of healthy controls, breast cancer patients and patients after chemotherapy. (**i**) The correlation between miR-200b and FUT4 levels in serum of healthy controls and breast cancer patients. *P<0.05, **P<0.01, ***P<0.001.

Our previous studies indicated that the upregulation of FUT4 was related to the proliferation and metastatic capability of some cancers.^32, 33^ Here we further analyzed differential FUT4 expression levels in breast cancer tissue microarray and found that FUT4 level was associated with the pathological stages of breast cancer (Table 1, Figure 1a). Next miR-200b and FUT4 levels were evaluated in the tissues and serum samples of breast cancer. We observed that miR-200b was lower (Figure 1b), whereas FUT4 were higher both in mRNA (Figure 1c) and protein levels (Figure 1e) in breast cancer than that in adjacent tissues. These data displayed a negative correlation between the expression levels of miR-200b and FUT4 (*r*=−0.8209; Figure 1d). Moreover, we collected serum samples from healthy women, breast cancer patients and those receiving regular chemotherapy to further analyze alterations in miR-200b and FUT4 by real-time PCR (Figures 1f and g) and enzyme-linked immunosorbent assay (Figure 1h). The results showed that, in the serum of breast cancer patients, miR-200b level was low, whereas FUT4 level was relatively high compared with that in controls, showing a negative correlation coefficient (*r*=−0.7581; Figure 1i). Serum sample outcomes were also in accordance with the results obtained in tissues of breast cancer. Further, the levels of miR-200b and FUT4 in the serum were recovered after chemotherapy, compared with those in untreated breast cancer patients. These results suggest that miR-200b and FUT4 are negatively correlated in breast cancer and may apply as novel biomarkers for the diagnosis and treatment evaluation.

### miR-200b inhibits proliferation, migration and invasion of MCF-7 and MDA-MB-231 breast cancer cells

**Figure 2:**
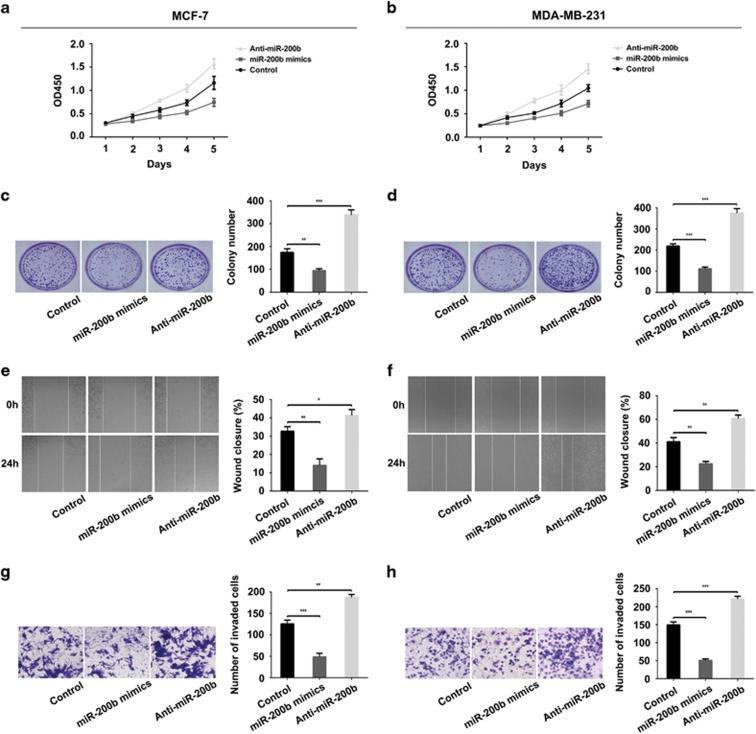
miR-200b inhibits the proliferation, migration and invasion of MCF-7 and MDA-MB-231 cells. (**a**, **b**) CCK-8 assay was applied to detect the ability of cell growth. Cells plated with a density of 3000 cells per well were transfected with miR-200b mimics and Anti-miR-200b, respectively. Absorption at 450 nm was detected for 5 consecutive days. (**c**, **d**) Colony-formation assay. Cells were plated with a density of 1000 cells per well after transfected with miR-200b mimics and Anti-miR-200b. Images were taken after surviving colonies were stained with crystal violet. The number of colonies was counted. Representative images and statistical analysis were shown. (**e**, **f**) Wound-healing assay. Cells were transfected with miR-200b mimics and Anti-miR-200b for 48 h and scratched with a 200 μl pipette tips. Then cells were cultured for another 24 h. The migratory ability of the cells was detected at the indicated times (0 and 24 h). Representative images and statistical analysis are shown. (**g**, **h**) Transwell assay. Cells (1 × 10^5^) were transfected with miR-200b mimics and Anti-miR-200b, respectively. Cells were added onto the upper chamber of the inserts followed by 24 h incubation. Invasion ability of the cells was determined after crystal violet stain. Ten fields in each group were recorded and counted per filter. *P<0.05, **P<0.01, ***P<0.001.

miR-200b expression showed high level in breast cancer tissues and the serum than in adjacent tissues or healthy people. Hence, we further explored the regulatory role of miR-200b in proliferation, migration and invasion of breast cancer cells. Cell Counting Kit-8 (CCK-8) assay and colony-formation assay were performed to assess the effect of miR-200b on the proliferation capability of cells after transfection and cultured for 7–10 days. CCK-8 assay indicated that miR-200b decreased the proliferation rate of breast cancer cells significantly when compared with the negative control, whereas cells transfected with Anti-miR-200b increased (Figures 2a and b). Similarly, colony-formation assay analysis revealed a decrease in colony number in the miR-200b mimics group and an increase in the Anti-miR-200b group than that in the control group (Figures 2c and d). We also investigated the potential effect of miR-200b on cell migration and invasion abilities. The results of wound-healing assay showed a lower rate of healing in the miR-200b mimics-transfected group, whereas Anti-miR-200b group showed a higher rate of healing than the untransfected group (Figures 2e and f). Similarly, transwell assay revealed that the invasion rate of breast cancer cells in the miR-200b mimics group was reduced compared with that of control, while miR-200b downregulation by transfection with Anti-miR-200b promoted the invasion significantly (Figures 2g and h). These results demonstrate that the proliferation and invasion potentials of breast cancer cells were inhibited by miR-200b.

### FUT4 is a novel target of miR-200b

**Figure 3:**
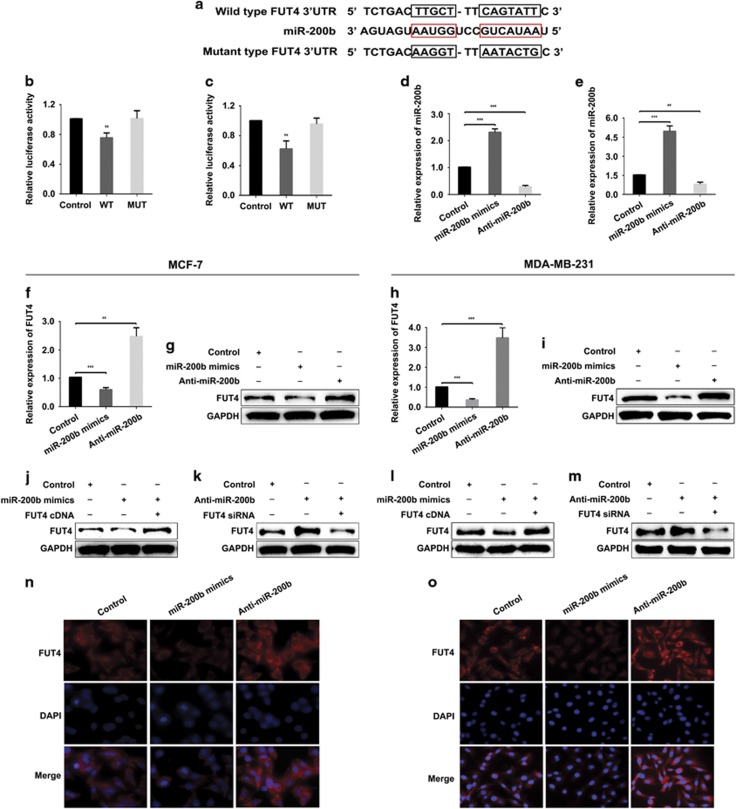
FUT4 is a novel and direct target gene for miR-200b. (**a**) A schematic showed reporter constructs of wild-type FUT4 3′-UTR (upper panel) and FUT4 3′-UTR with mutated miR-200b-binding sites (lower panel). (**b**, **c**) Dual luciferase gene report assay. MCF-7 and MDA-MB-231 cells were co-transfected with wild- or mutated-type reporter constructs and miR-200b mimics, respectively. Relative luciferase activity was detected by fluorescent intensity. WT: wild-type FUT4 3′-UTR transfection; MT: mutated-type FUT4 3′-UTR transfection. (**d**, **e**) Real-time PCR for the expression of miR-200b in MCF-7 and MDA-MB-231 cells after transfected with miR-200b mimics and Anti-miR-200b. (**f**, **g**) Real-time PCR and western blotting for FUT4 level in MCF-7 cells after miR-200b mimics and Anti-miR-200b transfection. (**h**, **i**) Real-time PCR and western blotting for FUT4 level in MDA-MB-231 cells after miR-200b mimics and Anti-miR-200b transfection. (**j**, **l**) Western blotting for the expression of FUT4 in MCF-7 and MDA-MB-231 cells after miR-200b mimics transfection or miR-200b mimics and FUT4 cDNA co-transfection, respectively. (**k**, **m**) Western blotting for the expression of FUT4 in MCF-7 and MDA-MB-231 cells after Anti-miR-200b transfection or Anti-miR-200b and FUT4 siRNA co-transfection, respectively. (**n**, **o**) Immunofluorescent staining of FUT4 in MCF-7 and MDA-MB-231 cells after miR-200b mimics and Anti-miR-200b transfection. Cells were immunostained with anti-FUT4 antibody (1:100) and exposed by TRITC goat anti-rabbit IgG secondary antibody (1:100). Representative images are shown by fluorescent microscope (magnification, × 400). **P<0.01, ***P<0.001.

Previous studies by our laboratory showed that FUT4 is an oncogene that promote cell growth and metastasis. Therefore, we attempted to identify potential miRNAs targeting FUT4. More than 10 databases were searched, including miRanda, TargetScan and PicTar. Among these, five databases predicted FUT4 as a potential target of miR-200b with highly matched base sequences or high scores between the 3′-UTR of FUT4 and miR-200b (Figure 3a). The data showed that the luciferase activity of FUT4 was reduced significantly after co-transfection of wild-type (WT) luciferase reporter constructs and miR-200b mimics, whereas there was little change in activity following co-transfection of MUT luciferase reporter constructs and miR-200b mimics in the two types of breast cancer cells (Figures 3b and c). Transfection efficiency was first evaluated by determining miR-200b level by real-time PCR after miR-200b mimics or Anti-miR-200b transfection (Figures 3d and e). In order to investigate the potential regulatory role of miR-200b on FUT4 gene and protein expression, real-time PCR (Figures 3f and h) and western blotting (Figures 3g and i) were performed. The data showed that miR-200b mimics significantly downregulated the FUT4 expression, whereas Anti-miR-200b induced FUT4 upregulation. These results were confirmed by co-transfection of miR-200b mimics and FUT4 cDNA (Figures 3j and l) and co-transfection of Anti-miR-200b and FUT4 small interfering RNA (siRNA; Figures 3k and m), which significantly restored or reduced FUT4 protein levels, respectively. Immunofluorescent staining of FUT4 revealed the similar changes (Figures 3n and o). The results suggest that miR-200b regulates the expression of FUT4 negatively via targeting its 3′-UTR directly.

### miR-200b inhibits the proliferation and invasion capabilities of breast cancer cells by suppressing FUT4 expression

**Figure 4:**
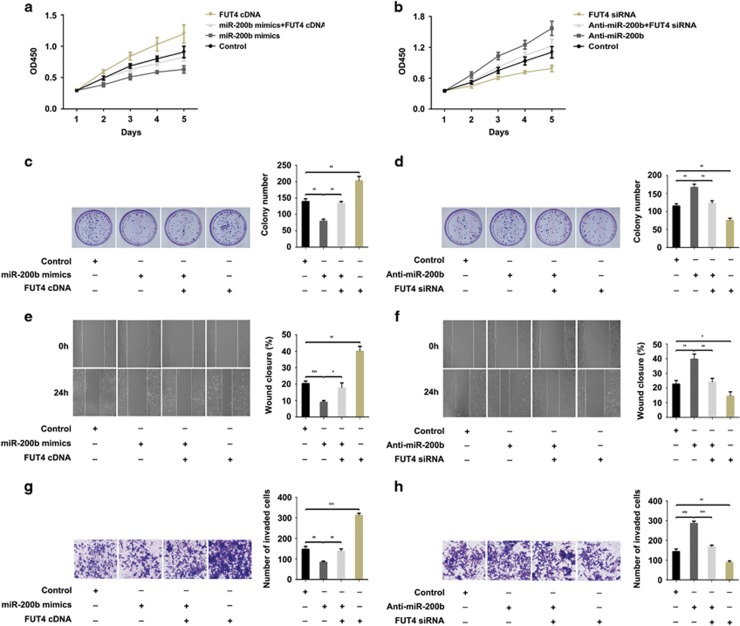
miR-200b inhibits the proliferation and invasion by suppressing the expression of FUT4 in MCF-7 cells. MCF-7 cells were transfected with miR-200b mimics and FUT4 cDNA or co-transfected with miR-200b mimics and FUT4 cDNA, respectively. (**a**) CCK-8 assay for cell proliferation. (**c**) Colony-formation assay. Representative images and statistical analysis are shown. (**e**) Wound-healing assay. Representative images and wound closure percentage are shown. (**g**) Transwell assay. Representative images and the number of invade cells were statistically analyzed. MCF-7 cells were transfected with Anti-miR-200b and FUT4 siRNA or co-transfected with Anti-miR-200b and FUT4 siRNA, respectively. (**b**) CCK-8 assay for cell proliferation. (**d**) Colony-formation assay. Representative images and statistical analysis are shown. (**f**) Wound-healing assay. Representative images and wound closure percentage are shown. (**h**) Transwell assay. Representative images and the number of invade cells were statistically analyzed. **P<0.01, ***P<0.001.

**Figure 5:**
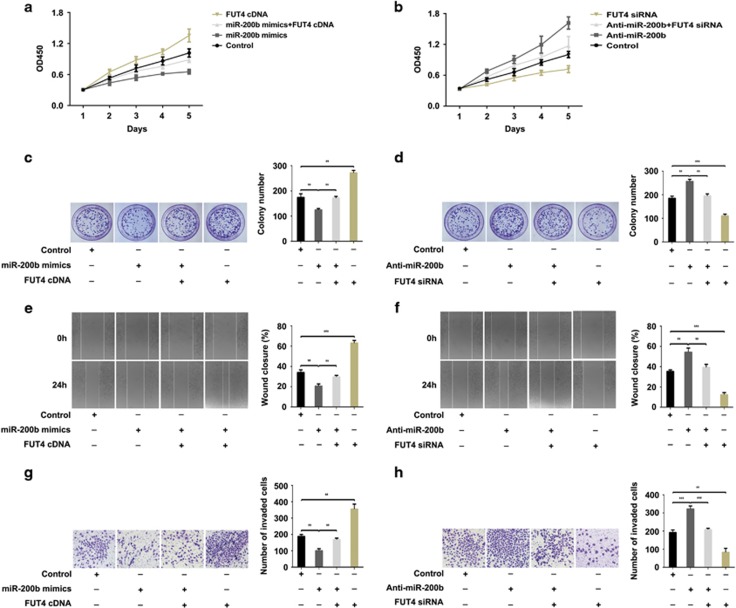
miR-200b inhibits the proliferation and invasion by suppressing FUT4 expression in MDA-MB-231 cells. MDA-MB-231 cells were transfected with miR-200b mimics and FUT4 cDNA or co-transfected with miR-200b mimics and FUT4 cDNA, respectively. (**a**) CCK-8 assay for cell proliferation. (**c**) Colony-formation assay. Representative images and statistical analysis are shown. (**e**) Wound-healing assay. Representative images and wound closure percentage are shown. (**g**) Transwell assay. Representative images and the number of invaded cells were statistically analyzed. MDA-MB-231 cells transfected with Anti-miR-200b and FUT4 siRNA or co-transfected with Anti-miR-200b and FUT4 siRNA, respectively. (**b**) CCK-8 assay for cell proliferation. (**d**) Colony-formation assay. Representative images and statistical analysis are shown. (**f**) Wound-healing assay. Representative images and wound closure percentage are shown. (**h**) Transwell assay. Representative images and the number of invade cells were statistically analyzed. **P<0.01, ***P<0.001.

To explore whether miR-200b exerts its function by regulating FUT4 expression, we prevented the expression of FUT4 in breast cancer cells of MCF-7 (Figure 4) and MDA-MB-231 (Figure 5) by miR-200b mimics transfection. Using CCK-8 and colony-formation assay, we found that cell proliferation was significantly reduced. Upon co-transfection of FUT4 cDNA and miR-200b mimics or FUT4 cDNA transfection alone, the proliferation rate was restored or increased significantly (Figures 4a and c, 5a and c). Additionally, we found that upregulation of FUT4 by Anti-miR-200b transfection promoted cell proliferation. In contrast, FUT4 siRNA co-transfected with Anti-miR-200b or FUT4 siRNA transfected alone in breast cancer cells significantly decreased the proliferation rate (Figures 4b and d; 5b and d). Further, by targeting the regulatory role played by miR-200b on cell migration and invasion by wound-healing assay (Figures 4e and f, Figures 5e and f) and transwell assay (Figures 4g and h, 5g and h) showed similar results. The data indicate that miR-200b inhibits the proliferation, migration and invasion of cells by targeting FUT4.

### miR-200b decreases α1,3-fucosylation of epidermal growth factor receptor (EGFR) and inactivates phosphoinositide-3 kinase (PI3K)/Akt signaling pathway

**Figure 6:**
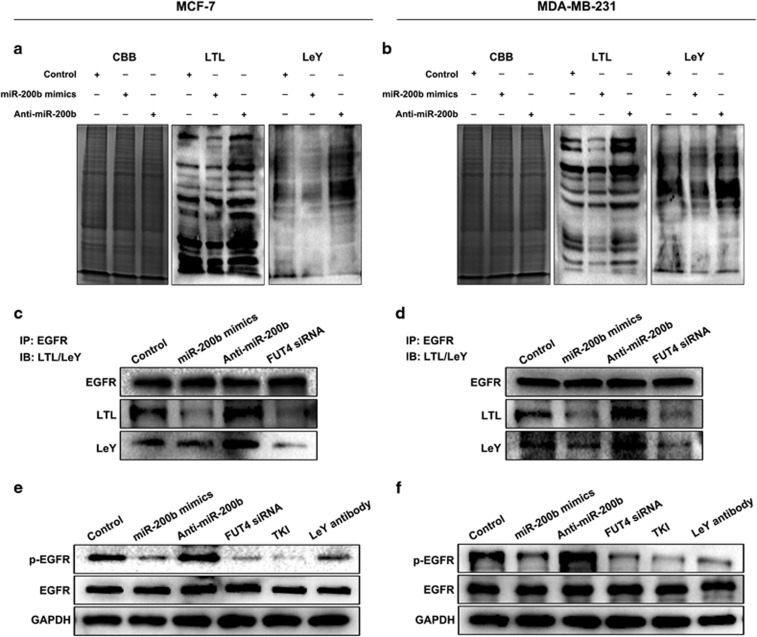
miR-200b decreases α1,3-fucosylation and phosphorylation of EGFR. (**a**, **b**) Fucosylation analysis by lectin blotting. Cells were transfected with miR-200b mimics and Anti-miR-200b, respectively. After 48 h culture, protein samples from cell lysates were harvested for detection. LTL lectin were used for detection of α1,3-fucosylation epitope and anti-LeY IgM antibody for assay of specific fucosylated antigen with α1,3-fucosylation linkage. CBB, Coomassie brilliant blue was applied as an equal loading control. (**c**, **d**) α1,3-Fucosylation of EGFR. Cells were treated with miR-200b mimics, Anti-miR-200b or FUT4 siRNA. Immunoprecipitation (IP): anti-EGFR antibody pulls down protein. Immune blot (IB): the level of α1,3-fucosylation was detected by LTL lectin and anti-LeY IgM antibody. (**e**, **f**) Phosphorylation of EGFR by western blotting. Cells were plated onto six-well plate and treated with miR-200b mimics, Anti-miR-200b, FUT4 siRNA, EGFR activation inhibitor (TKI) and anti-LeY IgM antibody (1:200), respectively. Anti-EGFR antibody (1:500) and anti-phosphorylated EGFR antibody (1:500) were used to detect EGFR activation.

EGFR is a scaffold carrying α1,3-fucosylation, particularly the LeY epitope.^34^ Based on the above results showing that FUT4 is a potential target for miR-200b that may regulate its expression, we hypothesized that miR-200b altered LeY synthesis and EGFR activation. The results of a blotting analysis showed that miR-200b mimics transfection induced a decreased expression of α1,3-fucosylation of glycoproteins by Lotus Tetragonolobus Lectin (LTL) (which specifically recognizes and binds to α1,3-fucosylated glycans) blotting analysis (Figures 6a and b), whereas Anti-miR-200b facilitated cell surface α1,3-fucosylation synthesis. LeY biosynthesis showed consistent changes (Figures 6a and b). After immunoprecipitation with EGFR antibody, the level of LeY on EGFR was further analyzed with either LTL lectin or LeY antibody. We found that transfection with miR-200b mimic reduced the LeY level on EGFR and silencing of FUT4 by specific siRNA inhibited LeY biosynthesis on EGFR, whereas Anti-miR-200b transfection induced an increase in LeY level (Figures 6c and d). These findings suggest that miR-200b prevents EGFR activation, whereas the Anti-miR-200b promotes its activation. An EGFR activation inhibitor (tyrosine kinase inhibitor) and LeY antibody as a negative regulator inhibited EGFR phosphorylation (Figures 6e and f). The results elucidate that miR-200b alters α1,3-fucosylation, LeY biosynthesis and LeY on EGFR by FUT4 downregulating FUT4.

**Figure 7:**
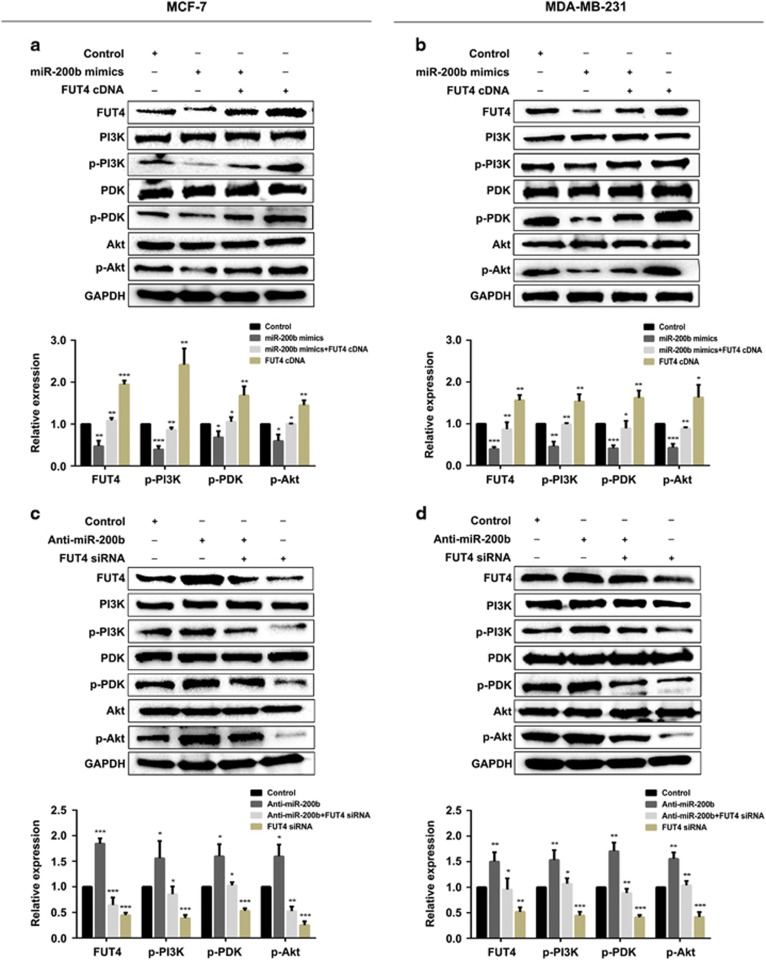
miR-200b inactivates PI3K/Akt signaling pathway through FUT4 downregulation. (**a**, **b**) Phosphorylation of PI3K, PDK and Akt was analyzed by western blotting and statistical analysis. Cells were transfected with miR-200b mimics and co-transfected with FUT4 cDNA or FUT4 cDNA alone, respectively. (**c**, **d**) Phosphorylation of PI3K, PDK and Akt was analyzed by western blotting and statistical analysis. Cells were transfected with Anti-miR-200b and co-transfected with FUT4 siRNA or FUT4 siRNA alone, respectively. *P<0.05, **P<0.01, ***P<0.001.

To further analyze the activation of downstream signaling pathway mediated by EGFR and induced by miR-200b, the signaling pathway of PI3K/Akt was evaluated. The results demonstrated that induction of miR-200b lead to p-PI3K, p-pyruvate dehydrogenase kinase (p-PDK) and p-Akt activation inhibition. Co-transfected FUT4 cDNA with miR-200b mimics or FUT4 cDNA transfected alone in cells significantly reversed or increased p-PI3K, p-PDK and p-Akt expression (Figures 7a and b). FUT4 upregulation by Anti-miR-200b transfection facilitated p-PI3K, p-PDK and p-Akt activation, whereas FUT4 siRNA co-transfected with Anti-miR-200b or FUT4 siRNA transfected alone significantly inhibited PI3K/Akt signaling pathway activation (Figures 7c and d).

### miR-200b inhibits tumorigenesis and metastasis of breast cancer *in vivo*

Based on the inhibitory role of miR-200b in tumorigenesis *in vitro*, we explored the suppression effect of miR-200b on tumor growth and metastasis *in vivo*, as well as its impact on the expression of FUT4 in xenografts. MDA-MB-231 cells were first injected into the mammary pad of nude mice for tumorigenesis, followed by different treatments.

**Figure 8:**
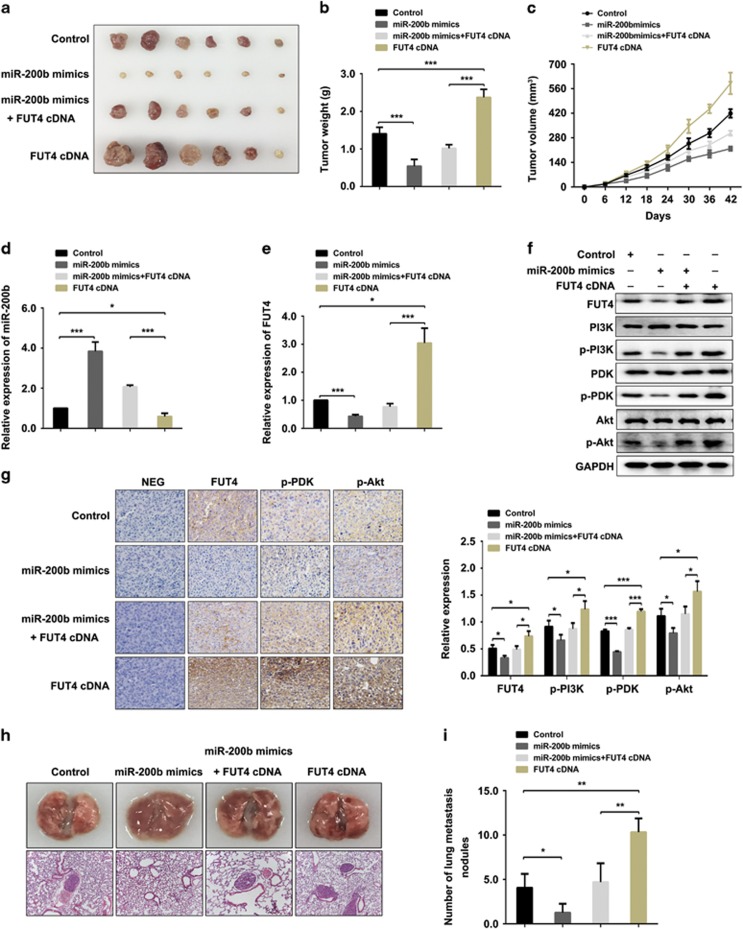
miR-200b inhibits the tumorigenesis and metastasis of breast cancer *in vivo*. MDA-MB-231 cells (5 × 10^6^) were injected into the mammary pad of female nude mice. Saline (control), miR-200b mimics, miR-200b mimics+FUT4 cDNA or FUT4 cDNA (100 μl per day) were injected for 6 consecutive weeks. (**a**) Pictures of xenografted tumors in control, miR-200b mimics, miR-200b mimics+FUT4 cDNA or FUT4 cDNA treatment group. (**b**, **c**) Tumor weight and volume were measured and statistically analyzed. (**d**, **e**) Levels of miR-200b and FUT4 in xenografted tumor tissues were evaluated by real-time PCR. (**f**) Protein samples were extracted and the expression of FUT4, phorsporylation of PI3K, PDK, Akt was analyzed by western blotting. (**g**) Tumor tissues were formalin fixed and paraffin-embedded sections were subjected to incubation with primary antibodies (FUT4, p-PDK, p-Akt). (**h**) Representative images of lung tissues (upper panel) and those of hematoxylin and eosin-stained lung sections showing the metastatic nodules. (**i**) The number of lung metastatic nodules of each group was analyzed. *P<0.05, **P<0.01, ***P<0.001.

We observed that miR-200b greatly impeded tumor growth, whereas FUT4 cDNA significantly promoted tumor growth in tumor volume and mass at the end point, compared with the control group. However, the tumorigenic function of FUT4 cDNA could be partly reversed by co-injection of miR-200b mimics and FUT4 cDNA (Figure 8a–c). The expression of miR-200b and FUT4, as well as the PI3K/Akt signaling pathway activation, in the xenografted tumor tissues were analyzed by using real-time PCR, western blotting and immunohistochemical staining. Results indicated that the expression of miR-200b was higher in the xenografts of the miR-200b mimics group than that in the control group (Figure 8d), whereas FUT4 expression in mice injected with miR-200b mimics was significantly decreased (Figure 8e). Meantime, PI3K/Akt signaling pathway was inactivated. Oppositely, FUT4 was upregulated and PI3K/Akt signaling pathway was activated in the FUT4 cDNA-injected group. Furthermore, the elevated FUT4 expression and activation of PI3K/Akt signaling pathway by FUT4 cDNA treatment could be partly reversed by co-injection of miR-200b mimics and FUT4 cDNA (Figures 8d and g).

The inhibitory effects of miR-200b on tumor metastasis was also determined. The number of metastasis nodules in lung tissues of the nude mice treated with miR-200b mimics was less by anatomical observation and hematoxylin and eosin staining, whereas that in the FUT4 cDNA-treated group were more than that of the control group. However, the enhanced metastatic potential by FUT4 cDNA treatment could be partly reversed by co-injection of miR-200b mimics and FUT4 cDNA (Figures 8h and i). These results highlight that miR-200b inhibit the tumorigenesis and metastasis of breast cancer cells to lungs via targeting FUT4 *in vivo*.

## Discussion

Specific circulating miRNAs are useful markers for the diagnosis, therapy evaluation and prognosis of breast cancer.^35, 36, 37^ miRNAs show different patterns of expression in the serum of breast cancer patients depending on their family subtypes or type of cancers.^38^ For example, Das *et al.*^39^ reported that the level of miR-720 was relatively high in the serum of both primary and metastatic breast cancer patients. Additionally, the levels of miR-103 and miR-107 in patient serum were potential predictors of the clinical benefit of endocrine therapy.^40^ Let-7b and miR-202 levels were elevated in the blood of breast cancer patients and correlated with tumor staging, whereas the expression of miR-34 was decreased significantly in the serum of breast cancer patients.^41^ To confirm the coincident changes of miR-200b in serum and tissues of patients, both serum and tissue samples were evaluated. We first found a significantly lower expression of miR-200b in tissues of breast cancer than that in paired adjacent tissues (Figure 1b), as well as a decreased level in serum of breast cancer patients compared with healthy women (Figure 1f). We further found that miR-200b level in the serum was recovered after chemotherapy, compared with that in untreated breast cancer patients (Figure 1f). Meantime, we also analyzed the FUT4 expression level and found that FUT4 was increased in patient serum and tissue samples (Figures 1c), indicating that FUT4 is a potential target for miR-200b. The level of miR-200b, as well as its oncogenic target, FUT4, in the serum are novel and specific diagnostic markers for breast cancer and are potential targets for breast cancer therapy.

miRNAs may function as antioncogenes to inhibit cancer proliferation and metastasis.^42^ It is reported that miR-200 family executed its regulatory roles by targeting different molecules, such as transcription factors, membrane receptors and cytoplasmic proteins. Fang *et al.*^43^ reported that ZEB2 modulated small cell lung cancer cells’ drug resistance that was regulated by miR-200b. Li *et al.*^44^ reported that miR-200b suppressed the proliferation, migration and invasion of osteosarcoma cells through inhibiting ZEB1 expression. For the essential roles of the members in miR-200 family on regulating EMT and the invasion of cancer cells via inhibiting the expression of ZEB1 and ZEB2, a study by Li *et al.*^44^ found that miR-200 repressed the metastasis of breast cancer through a moesin-dependent pathway.^45^ Sundararajan *et al.*^46^ found that TSK5 and MYLK, novel molecules in the formation of invadopodia, regulated the invasive behavior of breast cancer cells, which were mediated by the feedback loop of ZEB1/miR-200. However, the relationship between miR-200b and glycogenes remains unknown. Our previous study indicated that FUT4 promoted the proliferation and metastasis abilities of cancer cells, including breast cancer,^12^ non-small cell lung cancer^13^ and melanoma.^14^ Silencing of FUT4 by specific siRNA decreased the proliferation of A431 cells,^47^ and FUT4 inhibited epithelial–mesenchymal transition of MCF-7 and MDA-MB-231 cells.^32^ In this study, FUT4 expression level was correlated with the clinical stages of breast cancer in a tissue chip assay (Table 1, Figure 1a). Therefore, we hypothesized that inhibiting FUT4 was a potential strategy for inhibiting breast cancer development. To find a specific miRNA capable of targeting FUT4, we examined databases and predicted that FUT4 may be a novel target for miR-200b (Figure 3a). A luciferase reporter assay was used to confirm the specific binding between FUT4 and miR-200b (Figures 3b and c). The results revealed a significant suppression of FUT4 expression after transfection with miR-200b mimics, as well as decreased proliferation ability and invasion capability of breast cancer cells both *in vitro* and *in vivo* (Figures 4,5 and 8). Therefore, using clinical samples, breast cancer cells and breast cancer xenograft mouse model, we confirmed that FUT4 could serve as a new target gene for miR-200b.

We further investigated the putative mechanism of miR-200b targeting of FUT4 on glycosylation inhibition, as well as the suppression of cell proliferation and metastasis. Aberrant fucosylation is observed in many cancers,^48, 49^ and the inhibition of the fucosylation by the reduction of specific fucosyltransferases that are abnormally elevated may decrease tumor malignancy. Cheng *et al.*^50^ found that miR-26a, miR-34a and miR-455-3p negatively regulated the expression of FUT8 in hepatocellular carcinoma. Bernardi *et al.*^27^ reported that both miR-122 and miR-34a, which could target the 3′-UTR of FUT8 mRNA, were downregulated in the spontaneous hepatocarcinoma. FUT4 is responsible for the biosynthesis of α1,3-fucosylated glycans. Here we found that α1,3-fucosylation detected by LTL lectin blotting significantly decreased after FUT4 downregulation by miR-200b mimics transfection (Figures 6a and b). After confirmation of the whole α1,3-fucosylation alteration by miR-200b transfection, we further selected LeY as a representative of α1,3-fucosylated glycans to analyze the effect of miR-200b. LeY, which contains an α1,3-fucosylation epitope is carried by many glycoproteins, including EGFR,^34^ CD44^51^ and MUC6,^52^ on the cancer cells’ surface. EGFR is closely correlated with cancer cells’ proliferation and metastasis via the activation of EGFR and downstream signaling pathways.^53^ It has been reported that EGFR is overexpressed and activated in tissues and cells of breast cancer compared with normal control.^54^ LeY on EGFR may activate EGFR signaling pathway and facilitate the capabilities of proliferation, as well as the invasion, of many cancer cells.^14, 34, 55^ Functional blockage of LeY antigen by a specific anti-LeY antibody not only inhibited signaling of LeY to induce EGFR modification but also changed ErbB receptors in the SKBR-3 and A431 cells.^56^ Our previous work showed that silencing of FUT4 altered LeY biosynthesis followed by EGFR inactivation in A431 cells.^47^ Here we found that miR-200b and FUT4 siRNA transfection decreased LeY biosynthesis on EGFR (Figures 6c and d), which potentially inactivated EGFR (Figures 6e and f) and inhibited the PI3K/Akt signaling pathway (Figure 7), thus reducing proliferation and invasion potential of breast cancer cells (Figures 4 and 5). However, miR-200b is a small, stable and intrinsic molecule, with low side effects, and can more easily access target molecules; thus our findings contribute to the synthesis of new drugs for efficient EGFR inactivation in breast cancer cells and other LeY-EGFR-positive cancers. Furthermore, glycoproteins such as BMI1 may also contain an LeY epitope, thus miR-200b may possibly inhibit the function of these oncogenes. Further studies are needed to confirm these hypotheses.

In summary, our current study demonstrated that miR-200b level was lower in tissues and serum of breast cancer compared with that in normal controls. This study also emphasized that miR-200b targets FUT4 to negatively regulate its expression by decreasing α1,3-fucosylation, which may explain the inhibition of proliferation, migration and metastasis of breast cancer cells both *in vitro* and *in vivo*, and the inactivation of the PI3K/Akt signaling pathway. In conclusion, this study highlights a new strategy for gene therapy and diagnosis of breast cancer through a new network miR-200b/FUT4.

## Materials and methods

### Ethics statements

The clinical samples got the approval of the clinical ethics review board in the Second Hospital of Dalian Medical University. The procedures for animal experiments were performed in accordance to the guidance of Dalian Medical University for laboratory animals.

### Tissue samples

Twenty fresh-frozen tissues of breast cancer, as well as the adjacent non-cancerous tissues, were obtained from the Second Hospital of Dalian Medical University. Tissue microarray of breast cancer was purchased from US BIOMAX Inc. (Rockville, MD, USA). The samples were collected from the patients who did not receive any other treatments before surgery, such as chemotherapy and radiotherapy.

### Cell culture

MCF-7 and MDA-MB-231 breast cancer cells were obtained from ATCC (Manassas, VA, USA). Cells were cultured in Dulbecco’s modified Eagle’s medium/F12 containing with 10% calf serum and 1% Pen/Strep. All cells were maintained at 37 °C with 5% CO_2_.

### Transfection

Sequences used were as follows: FUT4 siRNA: 5′-GUUUGGAUGAACUUCGAGUTT-3′, 5′-ACUCGAAGUUCAUCCAAACTT-3′ miR-200b mimics: 5′-UAAUACUGCCUGGUAAUGAUGA-3′, 5′-AUCAUUACCAGGCAGUAUUAUU-3′ and Anti-miR-200b: 5′-UCAUCAUUACCAGGCAGUAUUA-3′. These sequences were synthesized in GenePharma Company (Shanghai, China). Transfection was conducted by using Lipofectamine 2000 reagent (Life Technologies, Graaand Island, NY, USA). The final concentrations of miR-200b mimics and Anti-miR-200b were 100 nM, while for FUT4 siRNA, it was 50 nM for 6 h. The samples were collected for gene and protein detection after 48 h.

### RNA extraction and real-time PCR

TRIzol reagent (TaKaRa, Tokyo, Japan) was applied to extract RNA from tissues and cells. The extracted RNA samples were used for reverse transcription. Real-time PCR was carried out on the StepOnePlus Real-time PCR System (Applied Biosystems, Life Technologies, Carlsbad, CA, USA) with SYBR Premix Ex Taq (TaKaRa). The sequences of primers were: miR-200b forward: 5′-TGCCGTAATACTGCCTGGTAA-3′, reverse: 5′-CAGAGCAGGGTCCGAGGTA-3′ U6 forward: 5′-ATTGGAACGATACAGAGAAGATT-3′, reverse: 5′-GGAACGCTTCACGAATTT G-3′ FUT4 forward: 5′-AAGGTCCAGGCCCAC TGAAG-3′, reverse: 5′-CAGTTCAGGTGACAGAGGCTCAA-3′ and GAPDH forward: 5′-ATGGGGAAGGTGAAGGTCG-3′, reverse: 5′-GGGGTCATTGATGGCAACAATA-3′. Each experiment was performed at least three times.

### Dual-luciferase reporter assay

Cells were co-transfected with 50 nM miR-200b mimics and 100 ng luciferase reporter constructs, including full 3′-UTR of FUT4 and mutation construct, in 24-well plates with Lipofectamine 2000 reagent. After transfection for 24 h, cells were lysed and the Firefly and Renilla luciferase activities were detected with the Steady-Glo Luciferase assay system (Promega, Madison, Wisconsin, USA). The luciferase activity of Firefly was normalized to the luciferase activity of Renilla.

### Western blotting

Cell proteins were harvested, and equal amounts of proteins were separated with 12% sodium dodecyl sulfate-polyacrylamide gel electrophoresis gel. Proteins on gels were transferred onto the nitrocellulose membranes, followed by 2 h of 5% fat-free dry milk blocking at room temperature. Primary antibodies were incubated overnight at 4 °C. After incubation with horseradish peroxidase-labeled secondary antibodies, ECL detection system was used to detect the immunoreactive proteins. The representative results from at least three independent experiments were shown. Densitometry of each protein band was quantified with the software of Image J (NIH, Bethesda, MD, USA).

### Cell proliferation assay

Cells were plated in 96-well plates with a density of 3000 cells per well and transfected with miR-200b mimics, Anti-miR-200b, FUT4 siRNA or FUT4 cDNA, respectively. CCK-8 (Beyotime Biotechnology, Biotechonology, Shanghai, China) solution was added that as followed by 2 h incubation. The absorbance at 450 nm was recorded for 5 consecutive days. Three independent experiments were conducted.

### Colony-formation assay

Cells were transfected with miR-200b mimics, Anti-miR-200b, FUT4 siRNA or FUT4 cDNA, respectively. After 10 days of transfection, the surviving colonies were fixed, followed by crystal violet staining. Images were taken under an inverted microscope (Olympus, Tokyo, Japan), and the number of colonies with >50 cells per colony was recorded.

### Immunofluorescent staining

After transfection, cells on the coverslips were fixed with 4% paraformaldehyde and permeabilized with 0.1% Triton X-100 at room temperature for 10 min. Serum (3%) was used to block the non-specific binding for 1 h. After blocking, cells were incubated with anti-FUT4 antibody (1:100) (Proteintech Group, Wuhan, China) at 4 °C overnight. After incubation with TRITC-conjugated goat anti-rabbit second antibody (1:100) (Proteintech Group) for 1 h at 37 °C and 4,6-diamidino-2-phenylindole for 3 min, images were acquired with a fluorescence microscope (Olympus).

### Immunohistochemistry

Tissue microarray of breast cancer, Paraffin sections and mouse xenograft tumors were deparaffinized and rehydrated followed by antigen retrieval in citrate buffer. After blocking with serum, FUT4 antibody (1:100), p-PDK (1:50), p-Akt (1:50) (Cell Signaling Technology, Danvers, USA) were incubated overnight at 4 °C. Then the tissue slides were incubated with anti-rabbit horseradish peroxidase-conjugated antibody for 45 min. The slides were stained and visualized by using 3,3′diaminobenzidine solution. Images were taken with microscope. The tissue section staining was analyzed blindly by at least two pathologists and cell scores were obtained. The expression level was divided into two categories: weak (cell score below average) and high (cell score above average).

### Wound-healing assay

After transfection with miR-200b mimics, Anti-miR-200b, FUT4 siRNA and FUT4 cDNA, respectively, cells were scratched with a pipette tip at 80–90% confluence and incubated for the indicated times. Images of cell migration were taken with an inverted microscope. The average extent of the wound closure for each group was quantified.

### Invasion assay

Transwell assay chambers (24 mm diameter, 8 μm pores, Corning, Corning, NY, USA) were coated with 50 μl Matrigel (1:8, BD Biosciences, San Jose, CA, USA) in 24-well plates for 4–6 h. After transfection, cells (1 × 10^5^) were placed in the upper chamber. The lower chamber was added with 800 μl culture medium containing 10% calf serum. After 24 h incubation, the chambers were fixed, and the cells were stained with crystal violet. The representative images were taken, and cells in 10 views were counted and analyzed. Triplicate experiments were performed for all experiments.

### Immunoprecipitation

Cell lysates were incubated with primary antibody against EGFR (Cell Signaling Technology) overnight at 4 °C. Immunocomplexes were purified by protein A/G agarose beads with gentle rocking. After washing for three times, immunocomplexes were resuspended in 20 μl sodium dodecyl sulfate loading buffer, followed by 70 °C incubation for 10 min and analyzed with western blotting.

### Tumorigenesis and metastasis assay *in vivo*

Female nude mice (4–6 weeks) were obtained from the Animal Center of Dalian Medical University (Dalian, China). The nude mice were randomly divided into four groups: control group (saline), miR-200b mimics group, miR-200b mimics+FUT4 cDNA group, and FUT4 cDNA group. MDA-MB-231 cells (5 × 10^6^) suspended in 100 μl saline were injected into the mammary pad of the mice. One week after injection, the mice were injected differently with saline, miR-200b mimics, mimics+FUT4 cDNA or FUT4 cDNA at the tumor cell inoculation sites in every other day. Tumor volume was measured and recorded every third day. The growth curves were made according to the tumor volume of each group. After 6 weeks of treatment, xenografted tumor tissues and metastatic lung tissues were harvested and the tumor weight was recorded. The lung tissues were dissected and stained with hematoxylin and eosin and observed under the microscope. The number of metastasis nodules was counted.

### Statistical analysis

All data gained from three independent experiments were represented as means±s.e.m. and analyzed with SPSS 17.0 (SPSS Inc., Chicago, IL, USA). Student’s *t*-tests were applied to analyze the differences between groups. Spearman’s correlation analysis was used to evaluate the relationship between miR-200b and FUT4. *P*<0.05 was considered to be statistically significant.

## Figures and Tables

**Table 1:** Clinical and pathological variables and the expression of FUT4 in breast cancer patients

*Clinicopathological factors*	*Number of cases*	*Expression of FUT4*		P*-value*
		*Low expression*	*High expression*	
*Age (years)*				0.754
<50	61	19 (31.1%)	42 (68.9%)	
>50	39	11 (28.2%)	28 (71.8%)	
				
*Degree of differentiation*				0.044*
Well and moderate	93	50 (53.8%)	43 (46.2%)	
Poor and signet	7	1 (14.3%)	6 (85.7%)	
				
*Clinical stage*				0.039*
I–II	82	35 (42.7%)	47 (57.3%)	
III–IV	18	3 (16.7%)	15 (83.3%)	

Abbreviation: FUT4, fucosyltransferase IV.
